# Metabolic, viral, and immune phenotypes in long COVID

**DOI:** 10.3389/fendo.2026.1899115

**Published:** 2026-08-11

**Authors:** Ivette F. Emery, Seynt Jiro Sahagun, Elizabeth R. M. Zunica, Christopher Axelrod, Robbie A. Beyl, Eunhan Cho, Guillaume Spielmann, Samantha Costa, Joanne T DeKay, Carolyn Chlebek, Shinya Amano, Katherine Stevens, Philipp E. Scherer, Dawei Bu, Aida Javidan, Brian S. Finlin, Xiaohua D. Zhang, Barbara Nikolajczyk, Catherine Blish C., Carolyn Bramante, Tracey McLaughlin, Soneida DeLine-Caballero, Kalani Ratnasiri, Uma Mangalanathan, Jonathan Z. Li, Brooke M. Leeman, Gregory Edelstein, Elizabeth W. Karlson, John P. Kirwan, Philip A Kern, Sergei Ryzhov, Clifford J Rosen

**Affiliations:** 1 MaineHealth Institute for Research, Scarborough, ME, United States; 2 Pennington Biomedical Research Institute, Baton Rouge, LA, United States; 3 Louisiana State University, Baton Rouge, LA, United States; 4 University of Pittsburgh, Pittsburgh, PA, United States; 5 University of Texas Southwestern, Dallas, TX, United States; 6 University of Kentucky, Lexington, KY, United States; 7 Stanford University School of Medicine, Stanford, CA, United States; 8 Department of General Internal Medicine, Harvard Medical School, Mass General Brigham, Boston, MA, United States; 9 University of Minnesota, Minneapolis, MN, United States

**Keywords:** glucose metabolism, glucose metabolism glycolysis, glycolysis, leptin, leptin T cells, T cells

## Abstract

**Context:**

Long COVID is characterized by persistent symptoms ≥ 3 months after acute SARS-CoV-2 infection. To date, the underlying pathophysiology is unclear.

**Objective:**

To characterize the immune and metabolic features of Long COVID and the potential role of viral persistence in adipose tissue.

**Design:**

Case-control, cross-sectional study under the RECOVER initiative.

**Setting:**

Maine, Louisiana, and Kentucky.

**Participants:**

Adults from the RECOVER study with high or low symptom burden assessed by the PROMIS scoring system or the Long COVID RECOVER Index (LCRI), matched by age, sex, BMI comparing post infected individuals with Long COVID vs those without sequelae.

**Main outcome measures:**

The primary outcome was a difference in T cell mitochondrial respiration by symptom severity. Secondary outcomes included glucose tolerance, body composition, T cell surface markers, subcutaneous adipose biopsy.

**Results:**

There were 54 participants, 80% female, mean age was 51.7 years, mean BMI 31. Time from initial infection was 894 days. The participant cohort by symptom burden was elucidated using a PROMIS symptom score subdivided by the following: High Symptom Burden (HSB) >15:n=25, Intermediate Symptom Burden (ISB) 10-15: n=14 and Low Symptom Burden (LSB) <10;n=15. Using the LCRI, n=15 were Long COVID+ (LC+) and n=39 were Indeterminate. The primary outcome, T-cell oxidative phosphorylation, did not differ between LC+ and Indeterminate nor by PROMIS scores. BMI and fat mass did not differ but T cell glycolytic activity was greater in those with LC+ vs Indeterminate. Lean mass and femoral BMD trended lower in the HSB vs LSB by both classifications. Prevalence of Type 2 diabetes did not differ by symptom scores, but HOMA-IR was higher and HOMA-B was lower in LC+ vs Indeterminate. SARS-CoV-2 viral RNA was not detectable in subcutaneous adipose tissue biopsies. CD26+ T cell number, and DPP-4 (CD26) activity were higher in the HSB and correlated significantly with symptom scores (r=0.52, p<0.01); plasma cytokines and stimulated T-cell cytokines did not differ by symptom group in either classification.

**Conclusions:**

Individuals with Long COVID symptoms show subtle impairments in glucose tolerance, serum leptin, lean mass and enhanced T-cell DPP-4 activity. Participants with Long COVID have subtle changes in glucose metabolism that are not driven by SARS-CoV-2 virus in subcutaneous adipose tissue.

## Introduction

Persistent symptoms beyond 90 days in individuals infected with the SARS-CoV-2 virus have been termed Post-Acute Sequelae of Covid-19 (PASC), or Long COVID (1). This disorder is manifested by a constellation of complaints, variable clinical signs and Indeterminate laboratory tests (2). Post exertional fatigue, pain, and dyspnea are the three most common complaints of individuals with Long COVID, and these appear to be worse among individuals previously hospitalized for acute SARS CoV-2 infections (3). Neurologic manifestations ranging from peripheral neuropathy to brain fog and mild cognitive dysfunction have been reported more frequently post infection with newer variants of SARS-CoV-2 (4). In that vein, the clinical course of Long COVID resembles to some degree myalgic encephalomyelitis/chronic fatigue syndrome (ME/CFS) (5). Overall, heterogeneity in the presentation of Long COVID as well as a host of possible pathophysiologic factors have led to considerable difficulty in identifying specific biomarkers for this condition.

The underlying pathophysiology of Long COVID has been tied to one or a combination of etiologic factors including viral persistence, immune dysregulation, endothelial disturbances with clotting disorders, and cardiometabolic dysfunction (6). One unifying hypothesis, linking several pathophysiologic elements, is chronic inflammation from metabolic dysfunction due to viral persistence. Ultimately, virus or viral remnants could provoke a chronic inflammatory state that worsens insulin resistance and/or drives persistent T cell fatigue. Obesity is a risk factor for acute COVID-19, and new onset glucose intolerance or Type II Diabetes (T2D) has been noted after acute SARS-CoV-2 infections (7, 8). An alternative thesis proposes that a leaky gut caused by dysbiosis from SARS-CoV-2 infection induces a chronic inflammatory state that is exacerbated by obesity and insulin resistance, as gut bacteria or their products enter the circulation and make their way to other tissues including adipose depots (9). Evidence to support viral persistence and a chronic inflammatory state includes a previous report that in acute COVID-19, SARS-CoV-2 RNA is present in adipocytes and the surrounding macrophages (10). Similar data from other fat depots were noted in small autopsy series early in the pandemic and in some animal studies (11, 12). In this study, named PROMIS, we sought to delineate the relationship between Long COVID symptoms and metabolic dysfunction and to investigate whether the latter was related to T cell dysfunction due to viral persistence in adipose tissue.

## Materials and methods (see also supplementary methods)

### Recruitment and consent

**Figure 1:**
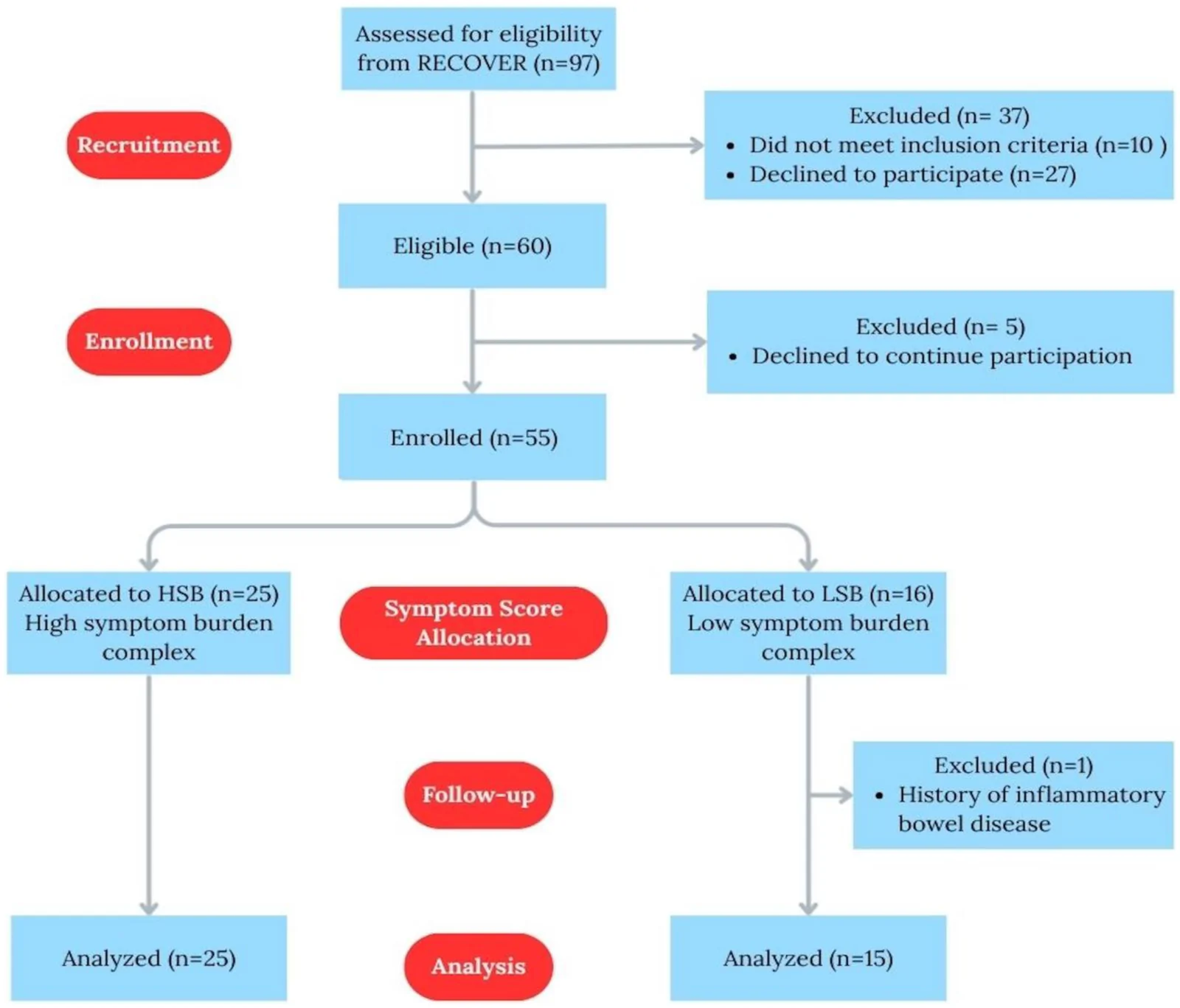
PROMIS consort flow diagram.

We initially targeted recruitment of sixty participants for this case-control study, PROMIS (Pathobiology in RECOVER of Metabolic and Immune Systems), from a pool of 97 individuals enrolled in the RECOVER observational study of Long COVID. Individuals were from rural and underserved areas of Maine, Louisiana and Kentucky (see **Figure 1**). Eligibility criteria included the following: 1) documented SARS-CoV-2 infection 90 or more days prior; 2) adults ages 21-75; 3) participation in the RECOVER-Adult cohort; 4) not involved in a clinical trial for Long COVID; 5) did not have Type I diabetes, 6) not pregnant, and 7) evidence that adipose biopsy would not carry unacceptable risk. This study was performed in accordance with the Declaration of Helsinki and Council for International Organizations of Medical Sciences International Ethical Guidelines and the International Council for Harmonization Good Clinical Practices Guidelines. All individuals signed informed consents at each of the three sites that was approved by a single institutional review board (IRB), the MaineHealth IRB, that also served as the single IRB during the conduct of the study. The study was performed between January 2023 and February 2025.

### Symptom scores and surveys

Initially we developed a patient-reported symptom score from questionnaires used in RECOVER prior to the LCRI that was published in 2024 (13). To assign individuals a symptom score that would correlate with the presence or absence of Long COVID at the time of enrollment, we examined responses from the most recent patient-reported RECOVER symptom survey completed more than 3 months after their initial SARS- breathless to leave the house or I am breathless when dressing or undressing”. CoV-2 infection and before enrollment in PROMIS. We added the number of symptoms for each participant indicated as “having now.” We then incorporated the severity of 8 key symptoms: shortness of breath, malaise, fatigue, faintness, brain fog, anxiety, pain, and poor sleep, by assigning a higher score for more severe symptoms. For example, in the description of shortness of breath, we added 1, if participant indicated “I get short of breath when hurrying on the level or walking up a slight hill,” 2 if participant indicated “I walk slower than people of the same age on the level because of breathlessness, or I have to stop for breath when walking on my own pace on the level,” 3 if participant indicated “I stop for breath after walking about 100 meters or after a few minutes on level,” and 4 if participant indicated “*I am too* In this manner, we generated an overall PROMIS symptom score that ranged from 0 (lowest) to 34(highest). Intermediate scores ranged from 10-15 (ISB). In comparison with the validated Long COVID Research Index (LCRI), the PROMIS scoring methodology incorporated the 4 key symptoms: shortness of breath, fatigue, malaise, and brain fog that are also found in the LCRI.

For the secondary analysis we grouped individuals into symptom categories using the LCRI which provides a score from a sum of symptoms. “Likely Long COVID, LC+ (with an LCRI >= 11)”, also referred to as PASC+ (13). The Indeterminate classification implies there are some elements of long COVID symptomatology whereas their composite scores did not reach the level of definitive Long COVID. Taken together the two scoring systems were similar and correlated closely (p<0.001) particularly for those with LC+ vs HSB, all analyses are presented for both systems although the intermediate symptom burden (ISB) group are also reported.

### Body composition by DEXA

A GE Lunar Prodigy Advance dual-energy X-light absorber (GE Medical System Lunar, Madison, WI, USA) was used for BMD measurements of the spine, femoral neck and whole body as well as body composition with an enCORE 2011 version 13.50.0 (GE Medical System Lunar, WI, USA) for scanning at the three sites: Louisiana, Kentucky, and Maine. During the scan, the participant remained in the supine position, with both arms on the body and palms on both sides facing down. All scans and adjustments were performed by trained technicians according to the International Society for Clinical Density Measurement (ISCD) Standards (14, 15). Body composition, including lean mass, fat mass, and ratios were assessed by DEXA. The DEXA analysis assumes a constant hydration of lean soft tissue, but hydration varies with age, gender and disease. The repeatability (CV) in the range of 1–2 percent body fat and 0.5–2 percent for lean tissue has been reported for DEXA. Using the ISCD BMD measurement accuracy calculation tool version 2.1 to calculate the accuracy of the whole-body data, the root-mean-square standard deviation (RMS-SD) and percent coefficient of variation (%CV) were determined to be 0.007 g/cm^2^ and 0.62%, respectively. The RMSD-SD and %CV used for the least significant change were 0.021 g/cm^2^ and 1.74%, respectively. Several of the participants also were scanned at the lumbar spine, and right and left femoral neck. Since all sites used the same DEXA instrument with a one-time measurement a single known standard was compared across sites to assess accuracy.

### Metabolic markers

In the RECOVER study, all participants who also entered PROMIS had non-fasting serum obtained and processed at the three local laboratories. These were reported in the same manner in the EPIC patient record, as well as in the RECOVER REDCap database. These included cortisol, HbA1c, cholesterol, HDL, LDL, and triglycerides. Adiponectin and leptin were measured by ELISA from fasting serum at MaineHealth Institute for Research after batching to include all samples from three sites (16, 17). Inter-assay coefficient of variation was 3.2% for adiponectin and 2.4% for leptin. Plasma endotrophin (ETP) levels were measured in the laboratory of Dr. Philipp Scherer at UT Southwestern. The coefficient of variation for the endotrophin ELISA assay was 4.9% with SD of 2.5 (18, 19). Bone turnover markers, CTx and P1NP, exploratory endpoints, were measured by ELISA from individuals in PROMIS at the Maine site only (n=20).

### Glucose and insulin studies

We measured glucose tolerance of the research participants using a standard 75g oral glucose tolerance test. Oral glucose tolerance tests (OGTT) were performed according to standard protocol: serum glucose, insulin, and c-peptide were measured at baseline, 30, 60 and 120 minutes after administering the glucose. Insulin and glucose levels from the OGTT were then used to determine HOMA-IR, HOMA-B, and the Matsuda index, which are measures of insulin resistance and sensitivity (20). We also determined the insulinogenic and disposition index, which are measures of beta cell function.

### Detection of virus in nasopharynx, adipose tissue, and blood

Quantification of SARS-CoV-2 viral load from nasopharyngeal (NP) swabs, plasma, and adipose tissue was performed using a previously described assay (21). NP swabs were placed in viral transport media (VTM), which was then aliquoted in 250 µL and stored at -80 °C until testing. VTM was thawed, homogenized, and pelleted at 25,200 x g for 2 hours at 4 °C. For plasma specimens, 300 µL of sample was mixed with 10 µL of replication-competent avian retrovirus (RCAS) virion as an internal quality control and centrifuged at 400 x g for 30 minutes at 4 °C to remove debris before being transferred to new tubes and pelleted at 25,200 x g for 2 hours at 4 °C (22). After pelleting, the supernatant was discarded, and 750 µL TRIzol-LS Reagent (ThermoFisher Scientific) was added and vortexed for 30 seconds. Following incubation on ice for 10 minutes, 200 µL of chloroform (MilliporeSigma) was added, and the mixtures were vortexed for 30 seconds. Phase separation was accomplished via centrifugation at 25,200 x g for 15 minutes at 4 °C. The aqueous RNA-containing layer was isolated and added to tubes containing 100 µL 3 M Sodium Acetate (Life Technologies) and 1.5 µL GlycoBlue Co-precipitant (ThermoFisher Scientific). 300 µL of Isopropanol (MilliporeSigma) was added and the mixtures were shaken, incubated in dry ice for 15 minutes, and then centrifuged at 25,200 x g for 45 minutes at 4 °C to precipitate RNA pellets. Afterwards the supernatant was discarded, and RNA pellets were washed with 900 µL cold 70% ethanol. RNA pellets were resuspended in 50 µL diethylpyrocarbonate-treated water (ThermoFisher Scientific) and used for qRT-PCR. The quantitative viral load reaction contained 5 µL extracted RNA, 1x TaqPath™ 1-Step RT-qPCR Master Mix, CG (ThermoFisher Scientific), and primers and probes amplifying the N1 region, n2019-nCoV (Integrated DNA Technologies). Absolute quantification of viral load was achieved via comparison to a standard curve generated by a serial dilution of N1 RNA with a 9 log10 dynamic range run on the same plate. The samples were analyzed on QuantStudio 3 real-time PCR systems (Applied Biosystems) using the following parameters: (stage 1) 2 minutes at 25 °C, (stage 2) 15 minutes at 50 °C for reverse transcription, followed by (stage 3) 2 minutes at 95 °C for initial denaturation, and (stage 4) 45 cycles of 3 seconds at 95 °C and 30 seconds at 55 °C. All plates contained two non-template control wells and a positive and negative control for N1. Positive control samples with 10,000 copies/mL were generated by extracting RNA from 50 µL of the AccuPlex™ SARS-CoV-2 Reference Material Kit (Seracare). Negative controls were made using 50 µL 10X PBS Buffer pH 7.4 (Corning). The efficiency of the RNA extraction and qRT-PCR amplification was evaluated by quantifying the RCAS RNA recovered from each plasma sample and the two N1 controls. Participant samples were run in triplicate wells for N1 and duplicate wells for RCAS. An aliquot of frozen RNA from adipose tissue was obtained from University of Kentucky, thawed, and evaluated using the same qRT-PCR protocol.

### Fresh Peripheral Blood Mononuclear Cells and Lymphocyte Studies

PBMC Isolation and Stimulation. Whole blood was collected into 8.5 mL acid citrate dextrose (ACD) tubes and gently mixed for 5 minutes at room temperature to incorporate preserving agents. Peripheral blood mononuclear cells (PBMCs) were isolated from whole blood by density gradient centrifugation using the Ficoll-Paque method. Red blood cells (RBC) were immunomagnetically depleted from the PBMC suspension by incubation with EasySep™ RBC Depletion Reagent and magnetic negative selection (Stem Cell Technologies). RBC-depleted PBMCs were then resuspended in warmed cell culture media (L-glutamine supplemented RPMI 1640) for downstream application.

One (1) mL of cell suspension (4-5x10^6^ cells per well) was plated into 4 wells of a non-tissue culture treated 24-well plate and incubated at 37 °C and 5% CO_2_ until the time of treatment. At the time of treatment, cells were exposed to one of the following conditions: 1) untreated control, 2) CD3/CD28 beads (1:1 bead to cell ratio), 3) SARS-CoV2 peptide mix (6 μL SARS-CoV2 PepTivator/0.5x10^6^ cells, Miltenyi Biotec), or 4) CD3/CD28 beads + SARS-CoV2 peptide mix. The plate was then incubated overnight at 37 °C and 5% CO_2_ for 16 hours.

Pan T cell Isolation. Following overnight stimulation, cell suspensions were collected and centrifuged at 350 x g for 8 minutes at room temperature. The pellet was resuspended in magnetic-activated cell sorting (MACS) buffer (1:3) (Miltenyi Biotec). Pan T cells were isolated by adding biotin-antibody cocktail (1:10) and incubated for 5 minutes in the refrigerator at 4 °C. MACS buffer (30uL/10^7^ total cells), and Pan T cell microbead cocktail (20uL/10^7^ total cells) were added and incubated for 10 minutes in the refrigerator (2-8 °C). The cell suspension was applied onto the column and the flow-through containing unlabeled cells was collected, representing the enriched T cell fraction. The isolated enriched Pan T cells were then centrifuged at 350 x g for 10 minutes at room temperature and resuspended with 1mL of RPMI media with 10% FBS. The cells were counted using an automated cell counter (Countess 3 FL; Thermo Fisher Scientific Inc.). The T cell purity was confirmed by incubating 1uL of anti-CD3 antibody fluorochrome for 20 minutes, protected from light at room temperature. A minimum purity of 95% was ensured by flow cytometry (BD Accuri C6). The isolated T cells were diluted to 1.5x10^6^ cells per mL in RPMI media with 10% FBS for preparation for respirometry.

### T cell surface markers, circulating cytokines, and *in vitro* T cell stimulation assays

Whole blood (8.0 mL) was collected in BD Vacutainer ACD tubes for n=50 subjects (MaineHealth n=20; UKY n=6; PBRC n=24). Upon receipt, blood aliquots (50µL) were obtained for flow cytometric analysis. Platelet-free plasma (PFP) was isolated at room temperature using 2-step centrifugation at 2,000g for 20 minutes. Aliquoted plasma was stored at -80 °C until further analysis. No more than one freeze/thaw cycle was permitted for PFP samples to avoid protein degradation. Peripheral blood mononuclear cells (PBMCs) were collected via Ficoll gradient protocol and stored at -80 °C for further analysis.

### Flow cytometric analysis

Blood aliquots underwent erythrocyte lysis with ammonium chloride lysing solution (150 mmol/L NH_4_Cl, 10 mmol/L NaHCO_3_, and 1 mmol/L EDTA, pH 7.4). White blood cells (WBC, 10^6^ cells/mL) were treated with whole molecule mouse and human IgGs to prevent nonspecific binding, followed by incubation with antibody panels for 25 minutes at 4 °C. Stained cells were washed once with 10 volumes of cold PBS/0.5% BSA/2 mM EDTA before data acquisition. Subpopulations of WBC were analyzed as described (REF) using the following antibodies: PE conjugated CD45 (H130), FITC-CD3 (UCHT1), PeCy7-CD8 (HIT8a), APC/Cy7-CD4 (OKT4), APC-CD26 (BA5b), PE-CD73 (AD2), FITC-HLA-DR (L243), PE-PD-1 (EH12.2H7), PE-CTLA4 (BNI3), PE-PD-L1 (29E.2A3), PeCy7-CD14 (HCD14), APC/Cy7-CD66b (G10F5), and APC-CD38 (HIT2) (BioLegend). An aliquot of blood was used to determine the absolute number of CD45^pos^ WBC using TruCount™ Tubes (BD Biosciences) according to the manufacturer’s protocol. The absolute values for cell subpopulations were calculated by multiplying the total number of WBC to the percent of events in the gate of interest. Viable and nonviable cells were distinguished with 4’,6-diamidino-2-phenylindole (DAPI) at the final concentration of 20ng/mL (72.5 nM). Data acquisition was performed on a MACSQuant Analyzer 10 (Miltenyi Biotec, Inc.); data were analyzed using WinList 5.0 and FlowJo 10.8 software (23).

### *In vitro* T cell cytokine stimulation assay

For the partial least square discriminant analysis (PLS-DA) cytokine data were log-transformed to mitigate skewing. Due to the properties of antibodies, amounts of different cytokines cannot be compared directly, and cytokines with a small range of values should have the option to be as or more important than cytokines with a large range of values. We thus scaled each cytokine to zero mean and unit variance before applying PLS-DA. After data preprocessing based on exploratory analysis, PLS-DA was applied mainly using the R function *splsda* in the R package “mixOmics” package^13^ and R^14^ version R4.2.2. Leave-one-out cross-validation (LOOCV) was adopted to mitigate overfitting and to enable a robust model selection and evaluation providing accuracies <70% (24–26).

### Statistics

The *a priori* statistical assessment plan was based on the case-control design, to compare individuals with HSB vs LSB by the PROMIS scoring system (**Figure 1**). The primary outcome was the difference by least square means in ATP generation from T cell oxidative phosphorylation (OXPHOS) between individuals with HSB versus LSB. It was powered on pilot data measured in fresh T cells by Seahorse, that showed ATP generation from OXPHOS of T cells was 30% lower in those previously infected with SARS-CoV-2 vs those not infected. The 95%confidence intervals for those differences between 10-50%. Thus, n=40 cases and 20 controls with a 30% suppression in OXPHOS would provide ≥ 99% power assuming a SD of oxidative phosphorylation measurements was 11%. A regression model was used to examine linearity across three symptom groups, HSB, ISB, and LSB using least square mean differences. Significant differences in the least square means were considered at a *p* ≤ 0.05. We did not adjust p values since this was an exploratory study.

A secondary analysis added subsequent to the publication of the Long COVID RECOVER Index (LCRI), was to compare individuals who were likely LC versus those who were considered Indeterminate using those scores (13). The correlation coefficient between scoring systems for all individuals in the cohort was r=0.65, p=0.01. Differences between symptom groups for all phenotypes was assessed by least square means either using the PROMIS categorization scoring system or the LCRI.

For the *in vitro* studies the partial least square discriminant analysis (PLS-DA) was used (27, 28). Cytokine data were log-transformed to mitigate skewing. Due to the properties of antibodies, amounts of different cytokines cannot be compared directly, and cytokines with a small range of values should have the option to be as or more important than cytokines with a large range of values. We thus scaled each cytokine to zero mean and unit variance before applying PLS-DA. After data preprocessing based on exploratory analysis, PLS-DA was applied mainly using the R function *splsda* in the R package and R version R4.2. For the metabolic studies, 2-way ANOVA, area under the curve determinations, data normality tests, Student’s t-tests, and Mann Whitney tests of data derived from the oral glucose tolerance tests were performed in GraphPad Prism 10.4.1.

For the bulk RNA-seq analysis, figures were generated in R using the ggplot2 package. Statistical analyses were performed as described in figure legend.

For the T-cell energetic studies, linear models were to estimate the mean effects of the groups. Least square differences of means were used to estimate the differences between groups. A sensitivity analysis was performed, which revealed the total T-cell yield as an independent covariate for energetic function. Thus, data were secondarily adjusted to account for gross differences in T-cell yield.

### Data availability statement

Raw sequencing data will be deposited under the NCBI Bioproject and processed data under GSEXXX upon publication. Analysis scripts will be available on github upon publication.

## Results

### PROMIS cohort

Ninety-seven individuals, all of whom were participants in the national RECOVER longitudinal study of Long COVID from sites in Maine, Louisiana, and Kentucky, were contacted for participation in the PROMIS study (see Consort Diagram **Figure 1**). Sixty women and men were eligible (n=60) by inclusion criteria, which included ages 18-75, participation in RECOVER, and previously confirmed SARS-COV-2 infection by reported PCR positivity, 90 or more days from enrollment. Exclusions included individuals with Type I diabetes, evidence that adipose biopsy may carry unacceptable risk, individuals <21 years of age and those with an autoimmune disorder or T2D. Five individuals withdrew consent (n=5) stating they were no longer interested in participating. Fifty-five individuals consented and completed (n=55) the study including body composition and T cell bioenergetics but one was eventually excluded from the low symptom burden group due to a pre-existing disease which was not disclosed initially and included chronic steroid use. The consort diagrams illustrate the classification of participants used by the two scoring systems (see **Figure 1**).

### Participant demographics in the PROMIS cohort

The PROMIS cohort characteristics including body composition are noted in **Table 1** and **Supplementary Table 1** by symptom complex classification. Eleven (20%) males and 44 (80%) females were enrolled; one male was excluded due to a significant co-morbidity and glucocorticoid use (see **Figure 1**). The mean time from initial SARS-CoV-2 infection to entrance in the study was 894 days (see **Table 1**). Mean age of participants was 51.7 ± 13.5 (SD) years, and mean BMI was 31.0 ± 6.5 (SD). Concomitant medications were variable across the groups, but anti-depressants were the most common, followed by cholesterol medications and vitamin D. One individual was discovered to have T2D while on the study, and did not have a GTT. Two participants were taking metformin but did not have T2D.

**Table 1:** Demographics by PROMIS Scores.

Characteristics	HSB (n=25) Mean ± Std	LSB (n=15) Mean ± Std	ISB (n=14) Mean ± Std	All (n=54) Mean ± Std
**Age**	51.88 ± 15.23	50.4 ± 13.56	52.79 ± 11.06	51.7 ± 13.58
Sex				
Female	21	10	11	42
Intersex	0	1	0	1
Male	4	3	3	10
Unknown	0	1	0	1
Race				
White	19	12	12	43
Black	2	1	2	5
Black/white	2	0	0	2
White/American Indian	1	0	0	1
White/Middle Eastern or North African	1	1	0	2
missing	0	1	0	1
Ethnicity				
Hispanic	2	0	0	2
Not Hispanic/Latino	23	15	14	52
Vaccination status				
Yes	23	14	13	50
No	2	1	1	4
**Time post infection, days**	1040.56 ± 301.99	783.67 ± 249.46	753.14 ± 184.53	894.69 ± 291.38
**Weight, lbs**	195.56 ± 42.04	172.49 ± 33.2	198.18 ± 46.14	189.83 ± 41.62
**BMI, kg/m^2^**	32.13 ± 6.78	28.65 ± 3.89	31.59 ± 7.53	31.02 ± 6.4
**% Fat Mass**	41.07 ± 10.31	38.89 ± 5.67	42.57 ± 9.47	40.86 ± 8.97
**% Lean Mass**	53.93 ± 5.14	58.39 ± 5.33	55.11 ± 8.85	55.48 ± 6.5
**Femoral BMD, g/cm^2^**	0.88 ± 0.12	0.97 ± 0.1	0.96 ± 0.17	0.93 ± 0.13
**Spine BMD, g/cm^2^**	1.15 ± 0.15	1.26 ± 0.14	1.16 ± 0.14	1.18 ± 0.15
**Whole Body BMD, g/cm^2^**	1.18 ± 0.15	1.19 ± 0.15	1.17 ± 0.14	1.18 ± 0.14

### Symptom scores in the PROMIS cohort

The PROMIS symptom scoring (subsequently called PROMIS score) revealed twenty-five participants who were considered to have symptoms of Long COVID (score>15) and were thus categorized as high symptom burden (HSB; n=25) (**Figure 1**). Fourteen participants had a PROMIS score between 10–15 and were categorized as intermediate symptom burden (ISB; n=14). Sixteen participants (with one individual eventually excluded), had few or no symptoms (score<10) and were categorized as low symptom burden or no symptoms (LSB; n=15) (See **Supplementary Table 1**, **Figure 1**).

Using the LCRI index in the PROMIS cohort, 15 participants were likely strongly positive for Long COVID (LC+, or PASC+), and 39 individuals were considered Indeterminate; i.e. intermediate scores that overlap to some degree with LC+. In that vein, twelve individuals in the Indeterminate range had PROMIS scores of >15, illustrating the differences between the scoring system (**Figure 1**). Overall, there were marked similarities between the two scoring systems and the correlation coefficient for the relationship between PROMIS scoring and the LCRI was r=0.65, p<0.01. A quarter of the cohort (n=15) had pronounced symptoms of Long COVID (termed high symptom burden, LC+) by the Long COVID Research Index (LCRI) while the other three fourths (n=39) had mild or residual symptoms and classified as Indeterminate (see **Table 2**).

**Table 2:** Clinical Phenotypes by LCRI Classification.

Phenotype	Likely Long COVID(LC)(n=15)	Indeterminate (n=39)	*P*-value (LC vs Indeterminate)
**Cholesterol, mg/dL**	220.53 ± 9.71	193.34 ± 6.10	0.02
**LDL cholesterol, mg/dL**	130.65 ± 8.76	106.46 ± 5.43	0.02
**Leptin, ng/mL**	41.0 ± 6.5	26.6 ± 4.14	0.08
**HOMA-B**	227.95 ± 47.60	101.21 ± 26.51	0.03
**HOMA-IR**	4.16 ± 0.85	2.36 ± 0.47	0.07
**Insulin, AUC**	12643 ± 1838	8708 ± 1098	0.07
**Left Femoral Neck BMD, g/cm^2^**	0.86 ± 0.04	0.95 ± 0.03	0.08
**CD14, cells/mm^3^**	0.81 ± 0.10	0.56 ± 0.07	0.05
**CD66b, cells/mm^3^**	0.81 ± 0.18	1.31 ± 0.13	0.03
**CD3/CD26**	59.48 ± 3.33	46.38 ± 2.32	0.002
**CD26 activity**	16.13 ± 1.43	9.94 ± 0.99	0.001
**Calcium, mg/dL**	9.67 ± 0.10	9.43 ± 0.06	0.04
**PTT**	32.27 ± 0.77	27.92 ± 0.48	<0.0001

Data presented as mean ± standard error.

The *a priori* statistical plan, based on the case-control design, was to compare individuals with HSB vs LSB by our PROMIS scoring system. However, based on the recent publication of the LCRI system, a secondary analysis compared individuals who were likely LC versus Indeterminate using the LCRI (13).

### Primary outcome: bioenergetics of T cells

No differences were observed in the oxygen consumption rates in T cells of participants with LC+ vs Indeterminate, or by the PROMIS scoring. across all experimental conditions (n=42). Extracellular acidification rates stimulated with CD3/CD28 beads or the SARS-CoV2 peptide mix were not statistically significant between LC+ participants relative to Indeterminate individuals. Uncoupled (UC) extracellular acidification (glycolytic) rates with the SARS-CoV2 peptide were participants classified as LC+ by the LCRI, vs Indeterminate (1.2 + 02. vs 0.6 + 0.2-; P=0.075) but did not reach statistical significance by PROMIS scores (p=0.18; **Table 3**).

**Table 3:** Clinical Phenotypes by PROMIS Classification with Effect Size.

Phenotype	HSB (n=25) Mean ±SE	LSB (n=15) Mean ±SE	*P*-value (HSB vs LSB) Mean ±SE	Effect size
**Leptin, ng/mL**	40.2 ± 4.8	18.3 ± 6.1	0.007	-0.343
**% Lean Mass**	53.93 ± 1.27	58.39 ± 1.64	0.035	0.290
**Spine BMD, g/cm^2^**	1.15 ± 0.04	1.26 ± 0.05	0.073	0.346
**R.Femoral BMD, g/cm^2^**	0.88 ± 0.03	0.99 ± 0.04	0.06	0.248
**Total protein, g/dL**	7.36 ± 0.08	7.09 ± 0.11	0.059	0.243
**WBC, cells x 10^3^/μl**	7.17 ± 0.35	5.79 ± 0.44	0.02	-0.293
**ETP, ng/mL**	39.14 ± 4.90	25.39 ± 6.54	0.099	-0.272
**Cortisol, *µ*g/dL**	8.79 ± 1.00	11.98 ± 1.46	0.08	0.326

### Secondary outcomes

#### Body composition and bone mineral density

Because the primary outcome of T cell oxidative phosphorylation was not statistically significant, the remaining outcomes are presented for hypothesis testing only. There were no statistically significant differences between participants with LC+ and those who were Indeterminate in respect to body weight or BMI except by PROMIS scores, whereby BMI in the HSB was significantly greater than in the low LSB (32.3 + 0.6 vs 28.1 + 0.70 P = 0.04). Percent lean mass differed between groups both by LCRI and PROMIS classifications (p=0.01 and p=0.005 respectively; **Table 2**; **Supplementary Table 2**). Fat mass in grams, by DEXA did not differ by group in either classification.

In respect to bone mass, whole body BMD did not differ by symptom group with either classification, but right femoral neck BMD tended lower in the LC+ vs Indeterminate (p=0.08), as well as by the PROMIS classification of HSB vs LSB (p=0.06). Spine and left femoral neck BMD were lower in those with high symptom burden but did not reach statistical significance(see **Table 2**, **Supplementary Table 2**). To assess the possibility of active bone loss, a subset (n=20) of the PROMIS cohort were tested for biochemical markers of bone turnover, and there were no differences between symptom groups in either C-terminal propeptide, CTx (LSB:0.33 ± 0.28 ng/ml vs HSB: 0.30 ± 0.14 ng/ml; *p* = 0.85), nor P1NP (LSB:56.2 ± 17.4 ng/ml; vs HSB:70.1 ± 32.0 ng/ml; *p* = 0.25).

#### Metabolic indices

**Figure 2:**
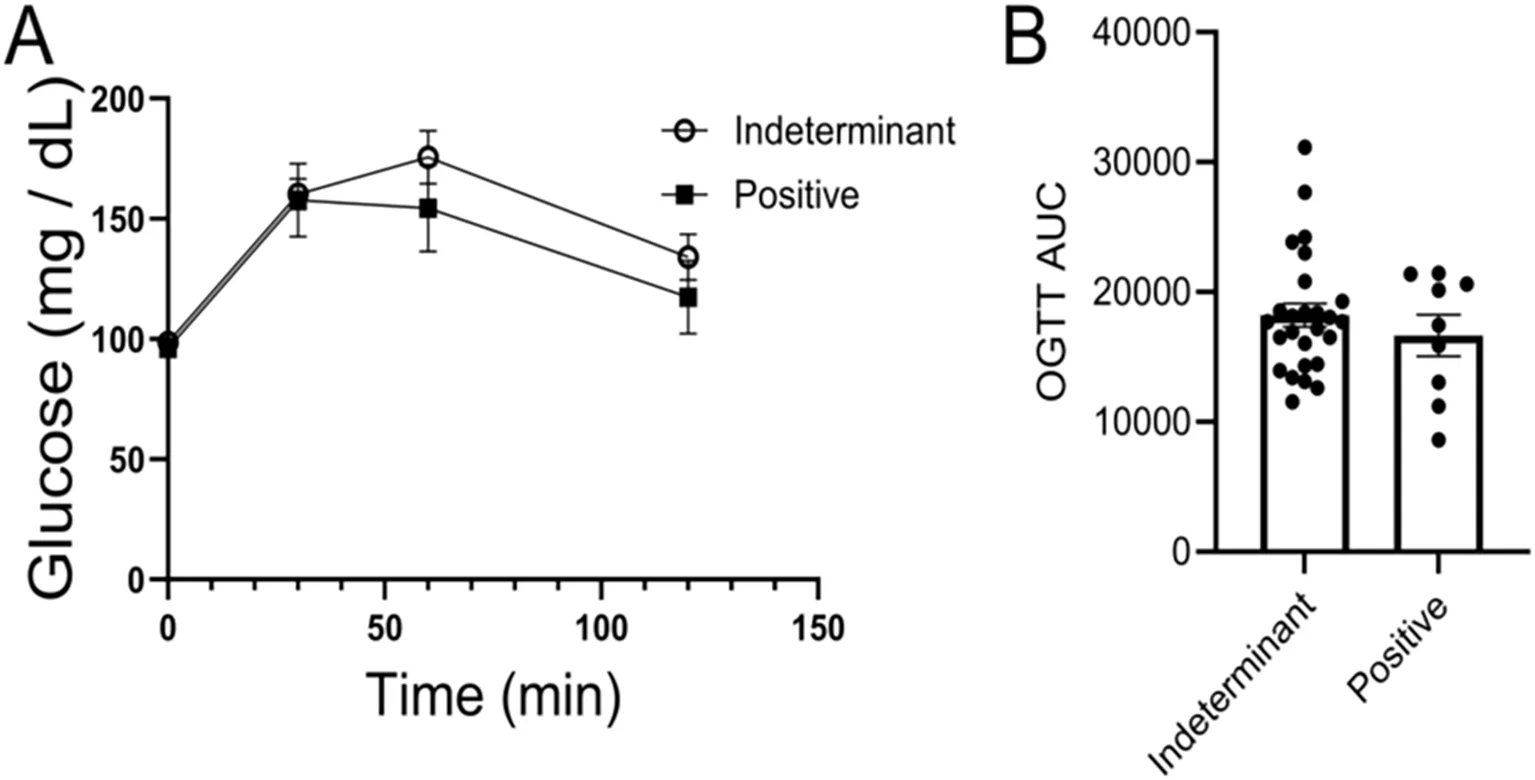
Glucose tolerance was not different between indeterminant (n=26) and long covid positive (HSB) (n=9) research participants. Standard 75 g oral glucose tolerance tests were performed. **(A)** Glucose was measured at 0, 30, 60, and 120 min. Data are presented as means ± SEM and were analyzed by 2-way repeated measures ANOVA (interaction: P = 0.53; long COVID status P = 0.41). **(B)** Area under the curve was calculated and analyzed by unpaired, 2-tailed Student’s t test.

Routine laboratory studies when classified by group with the LCRI, revealed higher total cholesterol (220.5 ± 9.7 vs 193.3 ± 6.1 mg/dL; *p* = 0.02) and LDL cholesterol (130.6 ± 8.8 vs 106.5 ± 5.4 mg/dL; *p* = 0.02) vs Indeterminate (see **Supplementary Table 2**) although neither were significantly different by PROMIS scores and effect sizes were relatively modest (see **Supplementary Tables 2A, B**). Fasting glucose, and glucose tolerance tests as well as the Matsuda Index did not differ by symptom group nor did HbA1C, CRP and d-dimer (see **Figure 2**) by either classification. But HOMA-IR was higher and HOMA-B was lower in the LC+ group vs Indeterminate participants (**Supplementary Table 4**). Leptin levels were higher in LC+ vs Indeterminate participants (26654 ± 4141 vs 40495 ± 6548 pg/mL; *p* = 0.08, see **Table 2**; **Supplementary Table 2**) and significantly higher in the HSB vs LSB by PROMIS scoring (p<0.007, see **Table 2**; **Supplementary Table 2A**). Adiponectin levels did not differ by group. Similarly, adiponectin: leptin ratios were not different when adjusted for BMI. Endotrophin levels were higher in those with heavy symptom burdens but not statistically different by LCRI 29.5+6.5 ng/ml; p=0.25) nor by PROMIS scores (HSB: 39.14+4.49 vs LSB: 25.4+ 6.2 ng/ml, p=0.099.

#### Adipose tissue SARS-CoV-2 Virus and adipose tissue gene expression

**Figure 3:**
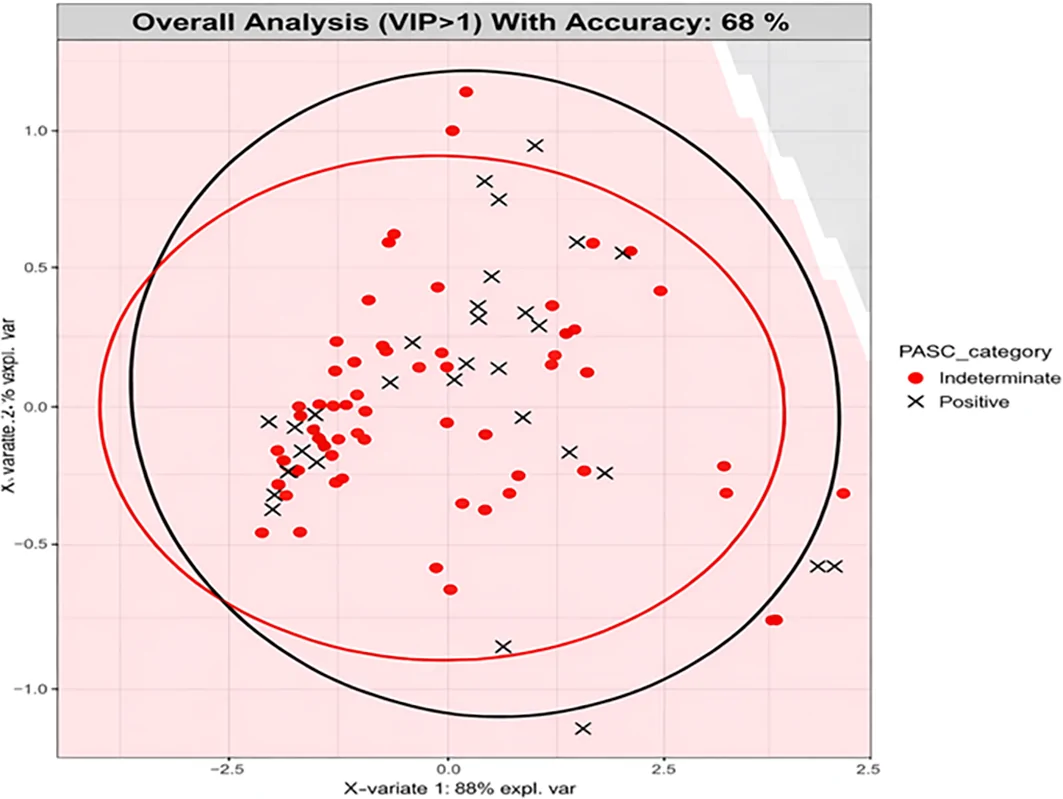
*In vitro* stimulated 25 cytokines (n=25) from T-cells using partial least squares discriminatory analysis. PLSDA was applied mainly using the R function *splsda* in the R package “mixOmics” package and R14version R4.2.2. Leave-one-out cross-validation (LOOCV) was adopted to mitigate overfitting and to enable a robust model selection and evaluation providing accuracies <70%. Overall performance was below the generally accepted cutoff of 70% for strongly predictive models.

No SARS-CoV-2 viral RNA or remnant was detected in any tissue compartment by qRT-PCR, including nasopharynx, blood, or subcutaneous adipose tissue. We next explored whether changes in gene transcription in subcutaneous adipose depots were associated with Long COVID symptoms to suggest there could be viral persistence. We compared participants with LC+ vs Indeterminate, first by unbiased bulk RNAseq, and then targeted qRT-PCR. Principal component analysis of all genes did not distinguish participants with LC+ vs Indeterminate. As there was evidence of a batch effect for samples sequenced at different times, we used MetaIntegrator to identify differentially expressed genes (DEGs) across the two batches. There were no significant DEGs HSB vs LSB or LC+ vs Indeterminate samples. Consistent with those negative findings, RNAseq failed to identify any evidence of viral RNA in the subcutaneous adipose tissue of 18 biopsies analyzed using the LCRI classification (see **Figure 3**).

In addition to bulk RNA-seq, we also performed qRT- PCR on fresh adipose tissue samples from 13 individuals in the Maine cohort. Among a panel of inflammatory markers, only IL-6, CRP, and Col6a3 mRNA were significantly increased, and IL-2 was significantly decreased in participants with LC+ vs Indeterminate (*p* < 0.05, see **Supplementary Figure 1**). There were no signs on histological evaluation of an active inflammatory process in adipose tissue. Similarly, we examined adipocyte inflammation by immunohistochemistry using CD68 and CD206 as markers for macrophage inflammation, density and/or polarity. We did not detect differences in macrophage type (pro- or anti-inflammatory) or relative quantity of staining. When we examined the secretome of fresh adipose tissue fragments that were cultured for 24 hours from individuals with LC+ vs those Indeterminate, there were no significant differences by symptom group (n=13).

#### T lymphocyte stimulation *in vitro*

**Figure 4:**
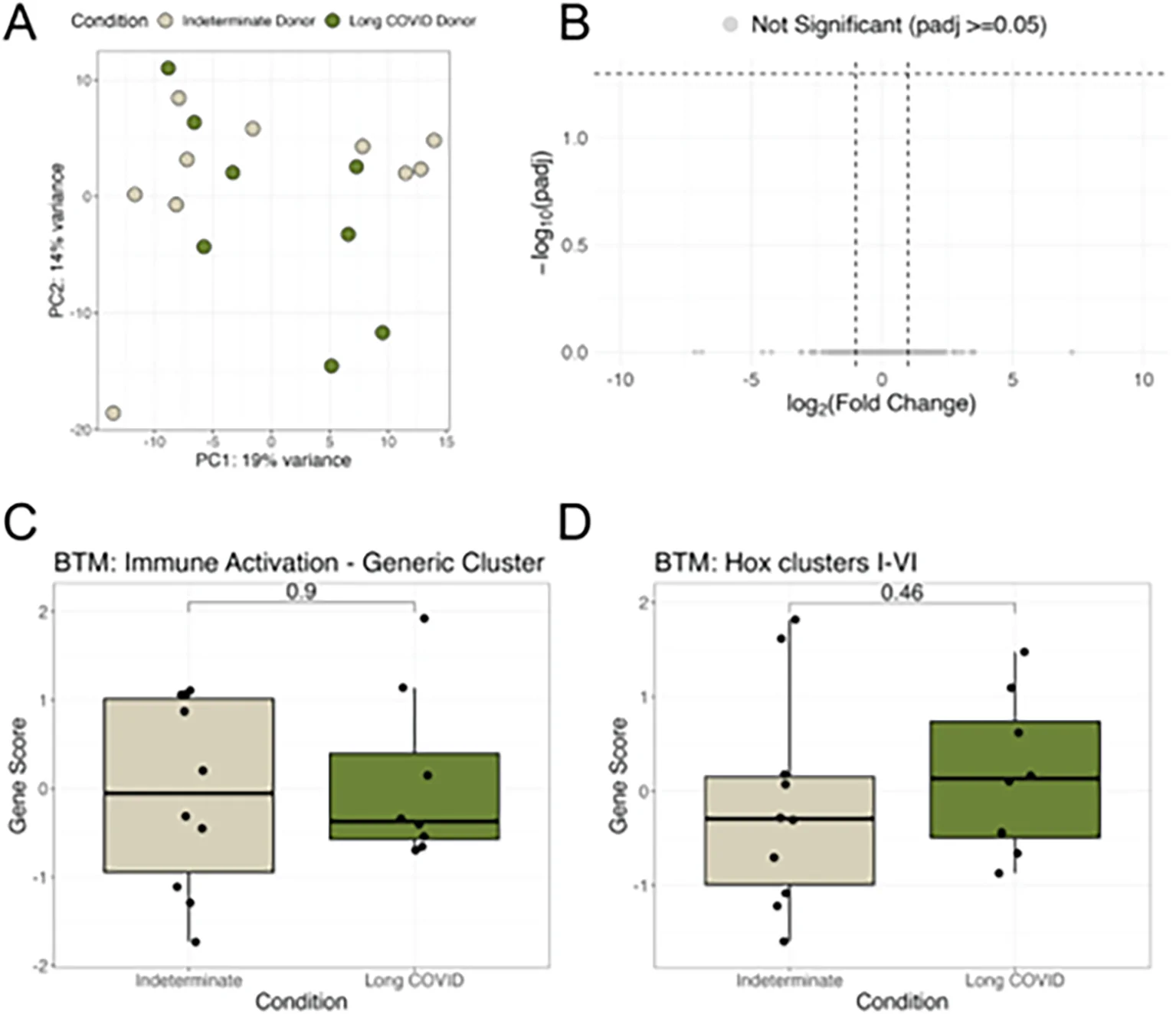
Long COVID (PASC+) is not associated with significant transcriptional changes compared to those with Indeterminate symptoms. **(A)** Dimensionality reduction by principal components analysis based on log_2_ normalized counts of all genes for participant samples. Each data point corresponds to the sample of one participant. PC1 principal component 1. PC2 principal component 2. Percentage expresses contribution to the overall data variability. **(B)** Volcano plot visualization of the differentially expressed genes between individuals with Long COVID symptoms and participants with Indeterminate symptoms. Horizontal dashed lines represent p adj p=0.05 and vertical dashed lines represent an absolute log fold change of 1. **(C, D)** Wilcoxon rank sum p values comparing gene scores between participants with Long COVID symptoms and those who were Indeterminate. For boxplot visualizations the top box line indicates the 75^th^ quartile gene score; middle box line indicates the gene score, and the bottom box line represents the 25^th^ quartile gene score.

There were no differences between groups in combinatorial cytokine quantities, using partial lease squares discriminant analysis models (see **Figure 4**).

#### T lymphocyte surface markers

Multiparametric flow cytometry analysis of freshly isolated leukocytes revealed no statistically significant differences between the major subpopulations of white blood cells between LC+ and Indeterminate participants (see **Table 4**; **Supplementary Table 2**; **Supplementary Figure 2**). The analysis of CD3+ T cell subsets demonstrated no increase in the percent and number of CD3/CD4 lymphocytes in LC+ participants; the frequency of CD3/CD8 cells did LC+ vs Indeterminate.

**Table 4:** Clinical Phenotypes by LCRI Classification with Effect Size.

Phenotype	Likely Long COVID(LC)(n=15)	Indeterminate (n=39)	Effect size
**Cholesterol, mg/dL**	220.53 ± 9.71	193.34 ± 6.10	-0.343
**LDL cholesterol, mg/dL**	130.65 ± 8.76	106.46 ± 5.43	-0.323
**Leptin, ng/mL**	40.50 ± 6.50	26.6 ± 4.14	-0.258
**Left Femoral Neck BMD, g/cm^2^**	0.86 ± 0.04	0.95 ± 0.03	0.303
**Calcium, mg/dL**	9.67 ± 0.10	9.43 ± 0.06	-0.289
**PTT**	32.27 ± 0.77	27.92 ± 0.48	-0.659

CD26 (DPP4) and CD73 (5’-nucleotidase) were used to evaluate CD4+ and CD8+ T cell activation and suppression. Four subsets were identified as CD26-expressing (CD26^pos^), CD26 and CD73 co-expressing (CD26/CD73^pos^), CD73-expressing (CD73^pos^), and CD26/CD73 double negative (CD26/CD73^neg^) cells. LC+ CD26+ CD4 T cells were not different from those in individuals who were Indeterminate. LC+ vs Indeterminate in levels of CD26- and CD73-expressing subsets of CD8 T cells. Similar non-significance was noted when participants were categorized by PROMIS scores.

The total pools of circulating CD26 and CD26/CD73 were also examined. The analysis showed a significant accumulation of CD26-expressing lymphocytes in the LC+ group vs Indeterminate (p=0.001) and also for CD3/CD26 ratios comparing LC+ vs Indeterminate (p=0.002) but not by PROMIS scores. There were no differences in the number of CD26/CD73 co-expressing T cells in LC+ vs Indeterminate groups nor by PROMIS classification. Finally, CD14 was lower in LC+ vs Indeterminate (p=0.05) and CD66 was higher in LC+ compared to Indeterminate (p=0.03) (**Table 4**).

#### Dipeptidyl peptidase activity in T cells

**Figure 5:**
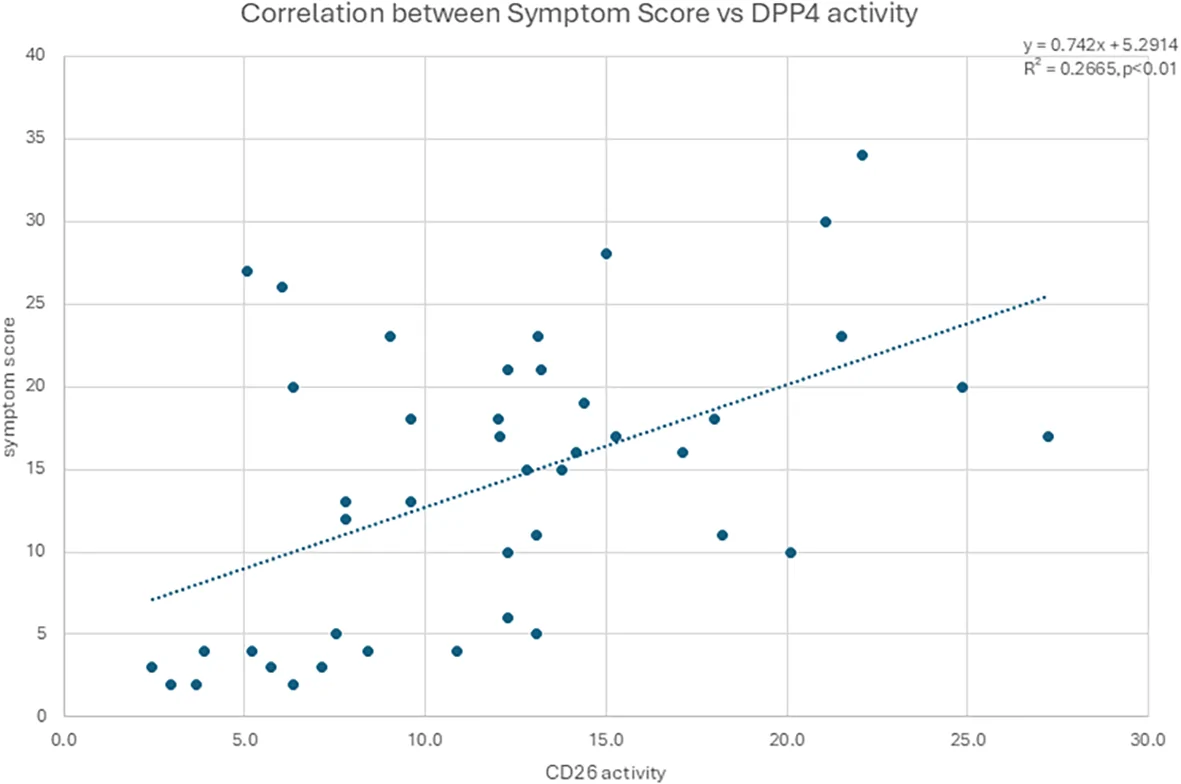
Symptom score correlation by PROMIS classification vs DPP-4 enzymatic activity.

DPP-4 enzyme activity was analyzed using frozen mononuclear cells. Flow cytometric analysis revealed a high correlation between the numbers of CD3/CD26 cells in freshly isolated leukocytes and frozen mononuclear cells, indicating that CD26-expressing T cells remained preserved after a freeze-thaw cycle. The analysis of the dose- and time-dependent effects of the CD26/DPP4 substrate, glycyl-L-proline 7-amido-4-methylcoumarine (GP-AMC) was used to choose the 100 μM concentration of GP-AMC and a 30 min incubation interval. The peptidyl peptidase activity was significantly higher in the mononuclear cell fraction obtained from LC+ vs Indeterminate groups (p=0.001). In addition, there was a strong positive linear correlation between symptom score from the PROMIS categorization and DPP-4 activity when adjusted for age and sex, r=0.52, y = 0.742x + 5.2914; p<0.01) (see **Figure 5**).

## Discussion

In a case-control study of RECOVER participants from Maine Kentucky and Louisiana we found that the primary outcome, T cell oxidative phosphorylation, was not impaired in those with confirmed Long COVID symptoms vs those who were considered Indeterminate. However, there were some alterations in T cell function. For example *in vitro* stimulated T cells with the SARS-CoV-2 peptide from individuals with LC+ had enhanced glycolytic activation, greater numbers of CD26+ T cells and increased dipeptidyl peptidase (CD26) activity which correlated closely with symptom burden. A previous study also demonstrated that individuals with Long COVID exhibited alterations in T cell subsets, including increased exhausted CD8+ T cells, reduced proliferative capacity of memory T cells, and a mis-coordination between T and B cell responses (29, 30). Other work has shown that mitochondrial function is impaired in T cells from individuals with Long COVID (31, 32). Notably, we report an increase in stimulated T cell extracellular acidification implying there may be a shift during activation of T cells, towards more rapid fuel utilization in symptomatic Long COVID participants. Nevertheless, the relatively small number of participants per group limit definitive conclusions about T cell fatigue and dysfunction.

In respect to body composition, lean mass but not fat mass differed by symptom group in the PROMIS cohort. The lack of differences in fat mass between groups was not unexpected since participants in each symptom group were originally matched by BMI, sex and age at enrollment. But, importantly, independent of fat mass, the high symptom group by each classification had greater leptin levels than those with few or any symptoms. Leptin is considered a proinflammatory adipokine. It shares structural and functional similarities with cytokines, directly activating immune cells (like T-cells and macrophages) and promoting the production of proinflammatory markers such as IL-6, TNF-α, and IL-1. As such, serum leptin, independent of fat mass, may be a marker of a low grade inflammatory response.

In respect to glucose tolerance, HOMA-IR (Homeostatic Model Assessment for Insulin Resistance), the glucose disposition index, and HOMA-B (Homeostatic Model Assessment of Beta-cell function) were different in the heavily symptomatic group compared to those with few symptoms. In particular lower HOMA-B in LC+ individuals suggest that in addition to mild insulin resistance, there may be impairment in beta cell release of insulin. These observations coupled with increases in CD26 T cell number and activity, suggesting enhanced breakdown of GLP-1, indicate aberrant glucose metabolism associated in the high symptom group. Moreover, there was a strong correlation between T cell CD26 enzyme activity and symptom scores across the entire cohort. Further studies are needed to assess the longitudinal nature of these subtle changes in HOMA-IR and HOMA-B, among Long COVID patients as to whether these are a prelude to T2D (33).

In respect to the possible pathophysiologic signals that lead to these changes, we were unable to detect overt inflammatory signals either in the circulation or locally. For example bulk RNA seq from subcutaneous adipose tissue failed to reveal any signal of inflammatory genes. Similarly, circulating cytokines did not differ by symptom group, nor did *in vitro* stimulation of cytokine release, or histologic analysis of adipocytes. In addition, although endotrophin levels, a marker of chronic fibrosis and inflammation, were higher in the LC+ group vs Indeterminate participants, these did not reach statistical significance.

Sarcopenic obesity has been proposed as a form of obesity characterized by low muscle mass and function with high BMI as well as indices of insulin resistance and hyperlipidemia (34–36). Since our cohort including both symptomatic and non-symptomatic individuals post COVID were all obese (mean BMI = 31), it is unlikely that obesity alone is pathogenic. But sarcopenic obesity could fit those individuals in the PROMIS cohort with a high symptom burden. They had reduced lean mass and lower bone mineral density than non-affected individuals. These characteristics could be a reflection of chronic frailty among individuals suffering from Long COVID, although other possibilities such as reduced physical activity or accelerated aging could be contributors. Taken together, individuals with Long COVID in the PROMIS cohort exhibited a multi-factorial combination of obesity, changes in T cell bioenergetics/function, subtle glucose intolerance, higher leptin levels and lower lean mass.

We were unable to detect SARS-CoV-2 in subcutaneous adipose tissue from any individual in the PROMIS cohort. Similarly, bulk RNAseq of adipose tissue failed to reveal major differences in immune transcriptomics between individuals with LC+ and those with few or no symptoms. In our proteomic analysis of conditioned media from cultured adipose tissue, we found no statistically significant differences between LC+ and Indeterminate individuals. Others have detected persistent virus in some individuals with Long COVID (37, 38). Even though we did not detect virus in subcutaneous tissue, it is conceivable that viral remnants could persist in other adipose depots (e.g. visceral fat) and could trigger a systemic or local inflammatory response. Consistent with that tenet, IL-6 and CRP mRNA expression were higher in subcutaneous adipose tissue from the symptomatic group than those with few or no symptoms suggesting there may be a local stimulus for an inflammatory response.

In contrast to the lack of evidence for overt adipose tissue inflammation, there were indications that a chronic systemic immune response might be present. CD26+ T cell frequency as noted previously, was higher in participants with LC vs Indeterminate. CD26 (aka DPP-4) is a multifunctional enzyme highly expressed in many tissues, that is directly involved in the activation of T cells as a co-stimulatory molecule (39). It possesses dipeptidyl peptidase activity and can cleave GLP-1. In that vein, enzymatic activity of dipeptidyl peptidase in mononuclear cells was found to be higher in those individuals with high symptom scores. Theoretically, high DPP-4 enzyme activity in T cells could also lead to greater degradation of GLP-1, which could explain the subtle changes in glucose tolerance we noted (39–41). Importantly there was a strong positive correlation between symptom scores from the PROMIS classification and CD26 (DPP-4) activity suggesting that further studies with larger populations could test whether T cell DPP-4 activity can predict active long COVID disease. Finally in analyses using the LCRI, we also found neutrophil activation of CD66b and CD14 positive cells, a potentially relevant interaction with the observed T cell changes. Taken together, our study suggests there is a dysregulation of CD4 T cell adaptive immune responses in Long COVID individuals who have a persistent symptom burden months after their initial infection.

The prolonged failure or inability of activated immune cells, driven by prior SARS-CoV-2 exposure, has been proposed as a pathophysiological mechanism of Long COVID (42). Our findings suggest that symptom burden–stratified differences in Long COVID are not driven by global impairments in mitochondrial oxidative capacity nor glycolysis in circulating T cells. However, we cannot exclude the possibility of metabolic *rewiring* during immune activation. The absence of differences in basal or stimulated oxygen consumption rates between individuals with LC+ versus Indeterminate indicates preserved mitochondrial respiratory competence, consistent with emerging reports that energetic fatigue in Long COVID does not uniformly reflect intrinsic mitochondrial failure (43). Instead, the selective elevation in uncoupled extracellular acidification rates in highly symptomatic individuals may point to a preferential reliance on glycolytic flux and inefficient energy transduction during T cell activation. More studies are needed to fully understand the bioenergetic component of T cell function in individuals with Long COVID.

There are several limitations to this case control study. First and foremost, our results suggest this study was underpowered for both primary and secondary outcomes. This might be related to differences between the LCRI and the PROMIS scoring system and our *a priori* prediction that 2/3 of the cohort recruited would have active Long COVID symptoms while a third would be symptom free. Second, this was a cross-sectional analysis. However, all the participants in PROMIS were also in the longitudinal RECOVER study and had questionnaire responses from time of first infection to entry in PROMIS. In fact, we found a very dynamic range of symptoms over 36 months in those individuals with high symptom burden, very consistent with recent RECOVER data that found that 5% had persistent symptomatology while 12% had non-resolving intermittent high burden symptoms (44, 45). Hence, it is likely that in PROMIS, most participants in the LC+ group were reflective of the group in RECOVER who had persistent or intermittent Long COVID symptoms. Third, long COVID is a heterogeneous disorder and other confounders certainly were likely at play, particularly recurrent asymptomatic SARS CoV-2 infections. Moreover, eight individuals did not complete all of the studies further lowering the likelihood to detect differences across symptom scores. In addition, among the intermittent group there were 14 individuals with scores in the mid-range (i.e. intermediate or ISB), which means these participants could be considered in either group, thereby further reducing our power (13). Fourth, the timing of sampling was relatively long after the initial infection (i.e.> 24 months); hence we could easily have missed subacute changes related to viral persistence in adipose tissue at earlier time points since infection or acquired immune responses reflecting ongoing inflammation. Another issue that could be relevant is the freshness of the T cells that were studied both for markers and for bioenergetics. In PROMIS the timing of testing most samples was relatively short (i.e.<4 hours), but some were shipped overnight from Maine and Kentucky and this alone could also have affected our results. Finally, we cannot exclude viral persistence at other sites in the body, including visceral adipose tissue, colon, or lung since these were not sampled.

In conclusion, in this case control sub-study of the RECOVER cohort we detected evidence of subtle changes in cardiometabolic indices and glucose tolerance in individuals with obesity and a high symptom burden, but no evidence of viral persistence in subcutaneous adipose tissue. These results suggest that a “sarcopenic obesity” type of phenotype could be evident in those with Long COVID symptoms. We also found subtle but significant enhancement in T cell glycolytic activity, increased CD26+ T cells, and greater DPP4 enzymatic activity that resembles to some degree ‘inflammaging’ with modest glucose intolerance (46). Larger and longer longitudinal studies are needed to validate these findings and define cardiometabolic risk in individuals with long COVID and obesity.

## Data Availability

The original contributions presented in the study are included in the article/**Supplementary Material**. Further inquiries can be directed to the corresponding author.

## Supplementary material

Supplementary Figure 1 Adipose Tissue Samples- Gene Expression; mRNA was measured by qRt-PCR in 13 samples after samples were frozen at -80C. PASC+ is HSB; PASC- is Indeterminate.

Supplementary Figure 2 Representative flow cytometry gating strategy. Peripheral blood aliquots (50 μl) were incubated with red blood cell lysis solution, resuspended in 0.5% BSA/2mM EDTA buffer, and stained with appropriate antibodies as described in the Methods section. **(A)** Representative dot plots showing the gating of major events and excluding potential marginal events with artificially high side and forward scatter characteristics (*left plot*, *black gate*), gating only viable DAPI-negative events (*middle plot, black gate*), and singlets to exclude cell aggregates (*right plot, blue gate*). **(B)** The percentage and number of CD45-positive events, representing white blood cells, were determined by gating on beads (BD Trucount™ tubes, black gate) and CD45-positive cells (*red gate*). Gates were established in the absence of beads and a CD45-specific antibody; *FMO*, fluorescence minus one. **(C)** Representative dot plot showing the appearance of beads in CD45-positive cells in corresponding gates. **(D)** Viable, singlets, CD45 immune cells gated as shown in Figures A–C, were used to determine the percentage of CD3 T cells. FMO control (*left plot*) was used to establish the positive gate for CD3 T cells (*right plot*). **(E)** Subsets of CD8 and CD4 T cells were determined within a subpopulation of viable, singlets, CD45-positive (CD45+) and CD3-expressing (CD3+) cells, using a gating strategy shown in Figures A–D. FMO controls for CD4 (*left plot*) and CD8 (*middle plot*) were used to establish gates for the CD4 and CD8 subsets (*right plot*). **(F)** Subsets of CD4-positive cells that express CD26 and CD73 alone, or co-express both CD26/CD73 at the cell surface, were determined within a viable, singlet, CD45+, CD3+, and CD4 - positive (CD4+) cells, using a gating strategy shown in Figures A–E. FMO controls for CD73 (*left plot*) and CD26 (*middle plot*) were used to establish gates for the CD26 and CD73 cells (*right plot*). **(G)** Subsets of CD8 - positive cells that express CD26 and CD73 alone, or co-express both CD26/CD73 were determined within a viable, singlet, CD45+, CD3+, and CD8 - positive (CD8+) cells, using a gating strategy shown in Figures A–E. FMO controls for CD73 (*left plot*) and CD26 (*middle plot*) were used to establish gates for the CD26 and CD73 cells (*right plot*).
