# Supplementary material for: Metabolic, viral, and immune phenotypes in long COVID
Source: Front Endocrinol (Lausanne). 2026 Aug 11;17:1899115. doi: 10.3389/fendo.2026.1899115 (PMC13508033; doi:10.3389/fendo.2026.1899115)
Supplement: Supplementary file 1 [file DataSheet1.pdf]

Supplementary Methods

Adipose Tissue Biopsy and Cultures

Adipose tissue collection was performed surgically by wedge biopsy (21). The patient was brought to the surgical operatory and prepped and draped in the usual manner. The area was marked with a surgical marker, anesthetized, and incised as drawn to the level of the subcutaneous fat with a #15 blade. Approximately 2 g of adipose tissue were removed with sharp scissor dissection. Hemostasis was maintained using spot electrodesiccation. The wound was closed with deep buried sutures of 4-0 Vicryl and reapproximated with interrupted sutures of 5-0 Prolene. The wound was then dressed with Steri-Strips, Tegaderm, and a non-adherent pressure bandage. The adipose tissue was divided into four pieces: three pieces in fixed buffered formalin; one piece flash frozen. One of the formalin fixed samples was transferred to 70% ethanol after 24-48 hours. While awaiting shipping, the formalin fixed samples were stored at room temperature, and the flash frozen sample was stored at -80°C. Fresh tissue samples of approximately 1 g were minced and placed in culture media for 24 hours according to an established protocol. Eighteen samples from Maine and Kentucky were ultimately collected by subcutaneous abdominal tissue biopsy with 2 g of tissue partitioned into three samples, one which was immediately flash frozen in -70ºC. Flash frozen samples were assayed for RT-PCR and bulk RNAseq. One set was kept in formalin for immunohistochemistry, and a fresh sample was cultured in conditioned media for 24 hours.

Bulk RNAseq of Adipose Tissue

Frozen samples from Maine were shipped for RNA extraction to the Kentucky site and stored with their samples. Approximately 240 mg of SAT was placed in a 2 mL tube with 700 mL Qiazol (#79306, Qiagen) and two 0.25 g stainless steel balls, and homogenized for 1 min at 1000 rpm in a SPEX sample prep 2010 Geno Grinder according to the manufacturer's instructions. After homogenization, the volume was adjusted to 1 ml with Qiazol, 200 mL chloroform was added, the 130 tubes were vortexed, incubated at room temperature for 5 min, and centrifuged at 12000 rpm for 15 min at 4ºC. RNA was the extracted from the aqueous phase 131 using a RNeasy kit (#74106, Qiagen) according to the manufacturer's instructions. Extracted RNA was subsequently shipped to Stanford University in the laboratory of Dr. Catherine Blish. At least 500 ng of RNA from each subcutaneous adipose tissue (SAT) sample was used for cDNA library preparation and bulk RNA sequencing, which was completed by GeneWiz/Azenta. RNA samples were diluted in nuclease-free water and tested to confirm a RNA Integrity Number (RIN) value of 6 or higher using the Agilent RNA ScreenTape assay and TapeStation system. NEBNext Ultra II RNA Library Prep Kit was used for cDNA library preparation. GeneWiz/Azenta completed sequencing with PolyA selection and generate approximately 20 million paired-end reads per sample using their Illumina NovaSeq 6000 platform (Illumina, San Diego, CA, USA). A quality report and FASTQ files were provided for further analysis.

Processing and analysis of bulk RNA-sequencing data

Raw fastq read files went through the following processing steps: FASTQC was used for assessing overall sample quality (22). TrimGalore was used to trim low quality reads

using the following parameters "--stringency 1 --gzip --length 20 --fastqc --paired" (23). Salmon was used for alignment to the human reference genome (GRCh38) using the default paired parameters(24). Tximport was used to import salmon alignment outputs, and DESeq2 was used for further processing (25,26). Variance stabilizing transformation normalization was performed (26). DESeq2 was used for Principal Component Analysis (PCA). MetaIntegrator was used for differential gene expression to take into account possible batch effects due to the two study sites(27). We utilized over-enrichment analysis to perform pathway analysis using the Blood Transcriptional Modules (BTMs)(28). We utilized published gene score methods to generate a per-sample score where we calculated the geometric mean of all genes in the specific BTM being analyzed, and scaling was performed across scores across all samples(29).

Cell-based Dipeptidyl Peptidase Activity Assay

The dipeptidyl peptidase activity in a fraction of PBMC was measured as previously described (36) with modifications. In brief, MNCs were quickly thawed and incubated in RPMI serum-free medium for an hour at 37°C in a CO_2 incubator to reduce inter-sample variability. Cells were washed, resuspended in PBS without Ca2+ and Mg2+, and added to a black 96-well plate with clear bottoms at a density of 10^5 cells/well. Then, 100 μL of Gly-Pro-AMC substrate at concentrations of 50, 100, and 200 μM in PBS was added. The fluorescence signals (excitation/emission wavelengths 350/450 nm) in each well were measured using a GloMax Explorer (Promega Corp.) every 10 minutes for up to 50 min.

Analysis of Circulating Cytokines: IL-8, IL-4, IL-6, and CCL11

Levels of interleukin-8 (IL-8), interleukin-4 (IL-4), interleukin-6 (IL-6), and C-C motif chemokine ligand 11 (CCL11) in platelet-free plasma were measured using corresponding ELISA kits per manufacturer protocols (Bio-techne/R&D Systems).

Oxygen Consumption and Extracellular Acidification Measurement of *T cells*. Mitochondrial function was determined in intact fresh cells by real-time respirometry (Seahorse XFe24; Agilent). Following isolation and dilution, 1 mL of 1.5×10^6 cells per mL were pelleted by centrifugation at 800 x *g* for 10 min at room temperature. The supernatant was removed, the pellet was gently washed with 500 mL of assay media (glucose-, pyruvate-, and phenol red-free DMEM supplemented with 2mM L-glutamine at pH 7.4 (Agilent)) and re-pelleted by centrifugation at 800 x *g* for 10 min at room temperature. Supernatant was fully removed (centrifugation was repeated if needed to fully remove supernatant) and pellet was resuspended in 305 mL of Assay Media. Cells were then evenly seeded at a density of 5.0×10^5 cells per well (100 mL of cell suspension) in a Cell-Tak coated (22.4 µg/mL) XFe24 well plate. The plate was centrifuged at 400 x *g* for 5 minutes at room temperature. After centrifugation cells were visualized under the microscope to ensure that they settled to the bottom of the well in an even monolayer. Additional assay media (DMEM + 2mM L-glutamine) was added to bring the final volume of each well to 500 µL. Each well was re-inspected to ensure that cell monolayer was not disrupted. Cells were then incubated at 37°C, 0% CO_2 for 1 hour prior to Seahorse assay.

Oxygen consumption rate (OCR) and extracellular acidification rate (ECAR) were then measured in real time using a Seahorse XFe24 analyzer (Agilent) before and after serial injection with D-glucose (25 mM final), oligomycin (1 µM final), FCCP or BAM15 (1-10

μM final), and rotenone/antimycin A/Hoechst (0.5/.05/2 μM final, respectively). OCR and ECAR were measured for three measurement cycles before addition of glucose and pyruvate and then measured for at least 3 measurement cycles after the addition of each compound (6 measurement cycles were used after FCCP/BAM15 in some experiments). ATP production was determined as the oligomycin sensitive rate. Electron transfer capacity was determined as the maximally uncoupled antimycin sensitive rate. Glycolytic capacity was defined as the maximal glucose-stimulated extracellular acidification rate corrected for non-glycolytic activity. Data were normalized to nuclear content by staining live cells after assay with Hoechst 33342 (ThermoFisher Scientific) and reading fluorescence on an automated microplate reader (Biotek) at excitation/emission 350/461 nm (31).

Proteomics from Cultured Adipose Tissue Biopsies

The conditioned media (CM) of cultured adipose fragments was concentrated to 150-200 μL, precipitated under 9 volumes of ethanol, isolated by centrifugation, and used for tryptic digestion as recently described. For unbiased protein identification, we used a data dependent acquisition (DDA) workflow. Nano-LC of tryptic peptides was performed on an UltiMate 3000 RSLCnano (Thermo Fisher, Waltham, MA). The separation column was fabricated in-house (ReproSil-Pur C18-AQ, 5 μm particle, 120 Å pore column of dimensions 50 μm I.D. × 15 cm length. Gradients were performed with Burdick & Jackson LC-MS-grade solvents (Honeywell, Morris Plains, NJ) with Optima-grade formic acid (Fisher Chemical, Pittsburgh, PA). Channel A pumped 0.1% formic acid, while channel B ran 0.1% formic acid in ACN. The separation column was equilibrated

at 45 °C with 4% B at 300 nL/min. Flow rate was increased to 350 nL/min for sample loading. In all cases, approximately 1 µg of sample was loaded via the autosampler fitted with a 3.6 µL silica capillary sample loop. After 10 minutes of loading, the loop was taken out of the flow path and flow rate returned to 300 nL/min. A linear gradient to 30% B at 95 minutes was then executed, followed by a gradient to 50% B at 113 minutes, then a rapid gradient to 92% B at 115 minutes. Washing was performed to a run time of 120 minutes, at which point the system was returned to starting conditions and equilibration performed for 10 minutes before the next sample was run. Column eluate was directed through a stainless-steel union into a Sciec 5600 mass spectrometer via a silica emitter (10 µM I.D. PicoTip, New Objective, Littleton, MA), which interfaced the mass spectrometer (TripleTOF 5600, Sciex, Framingham, MA) via a Nanospray III source operating at 2600 V and 150°C employing a nitrogen sheath gas at 14 psi and curtain gas running at 23 L/min. Protein identification was conducted using two replicate LC-MS runs evaluated using the Paragon algorithm in the ProteinPilot software package (Sciex, Version 5.0.2) to search a Uniprot reviewed human database with a <5% false discovery rate (FDR) at the peptide level (33).

Cultured Adipose Tissue Samples

Human subcutaneous adipose tissue (SAT) obtained by full biopsy included 2 g of tissue being partitioned into three samples, one of which was used for adipose tissue cultures. For each culture setup, adipose tissue from one subject was used. Adipose tissue biopsies were transported from the operating room to the laboratory in 50 mL of sterile transport buffer (PBS, 10% FBS, and 5% penicillin/streptomycin) on ice. The following procedures were carried out under a laminar flow hood using sterile

equipment. Immediately upon arrival, the tissue was transferred to a 100 mm Petri dish containing 10 mL of room temperature (RT) PBS and was finely minced into 20–80 mg pieces using scissors and/or scalpels. The tissue pieces were washed with 400 mL of 4 °C PBS over a 40 mm nylon filter. Afterward, the adipose tissue pieces were transferred to a 75-cm^2 culture flask containing 200 mL of RT PBS and were gently shaken for about 5 minutes. Next, the contents of the flask were poured over the filter, and the tissue pieces were washed with 300 mL of pre-warmed PBS (37 °C). The tissue pieces were transferred to a tube containing 50 mL of RT PBS and centrifuged for 1 min at 277 x g at room temperature to remove red blood cells and debris. The floating adipose tissue was removed from the tube and then placed in a 100 mm Petri dish with 15 mL aMEM culture medium containing Earle's Salts and supplemented with 2% penicillin/streptomycin (no FBS was used in the culture media). Adipose tissue pieces were cultured at 37 °C and 5% CO_2. The conditioned medium was collected after 1-, 2-, 16-, and 48 hours. This removed adipose tissue metabolites and secretory proteins, which accumulated during the culture. After each collection, the culture medium was replaced with 15 mL of fresh media. The final secretome samples within the conditioned media (CM) were frozen at -20 °C for 24 hours, then stored at -80 °C. After 48 hours, the remaining adipose tissue was washed with PBS, then flash frozen and stored at -80 °C. This method was based on the adipose culture protocol found in Alvarez-Llamas et al, 2007 (32).

Immunohistochemistry of Adipose Samples

We performed immunohistochemistry staining on a subset of SAT samples from both HSB and LSB groups (Supplemental Table 2). For this staining, a portion of adipose

tissue was fixed in formalin, embedded in paraffin, and sectioned. Five mm sections were co-stained with CD86 and CD68 antibodies or CD206 and CD68 antibodies as described (34).

Transcriptomics of Cultured Adipose Tissue

Total RNA was extracted from the cultured adipose tissue using TRIzol (Thermo Fischer Scientific). A total of 500 ng of RNA was converted to cDNA (High-Capacity cDNA Kit; Invitrogen, 4368813), then diluted 1:10. Quantification of mRNA expression was carried out via quantitative RT-PCR using AzuraQuant Green Fast qPCR Mix (Azura Genomics, AZ-2120), and thermocycling reactions were completed using a CFX-384 (Bio-Rad Laboratories). Data were analyzed using Bio-Rad CFX Manager 3.1 and Excel using the delta-delta CT method. Technical duplicates were run, then averaged for each biological data point. TATA-binding protein (TBP) was used as the housekeeping gene. A list of genes can be found in the Supplemental Material (Supplement Table 1).
