# Supplementary material for: Metabolic, viral, and immune phenotypes in long COVID
Source: Front Endocrinol (Lausanne). 2026 Aug 11;17:1899115. doi: 10.3389/fendo.2026.1899115 (PMC13508033; doi:10.3389/fendo.2026.1899115)

Supplemental Figure 1.

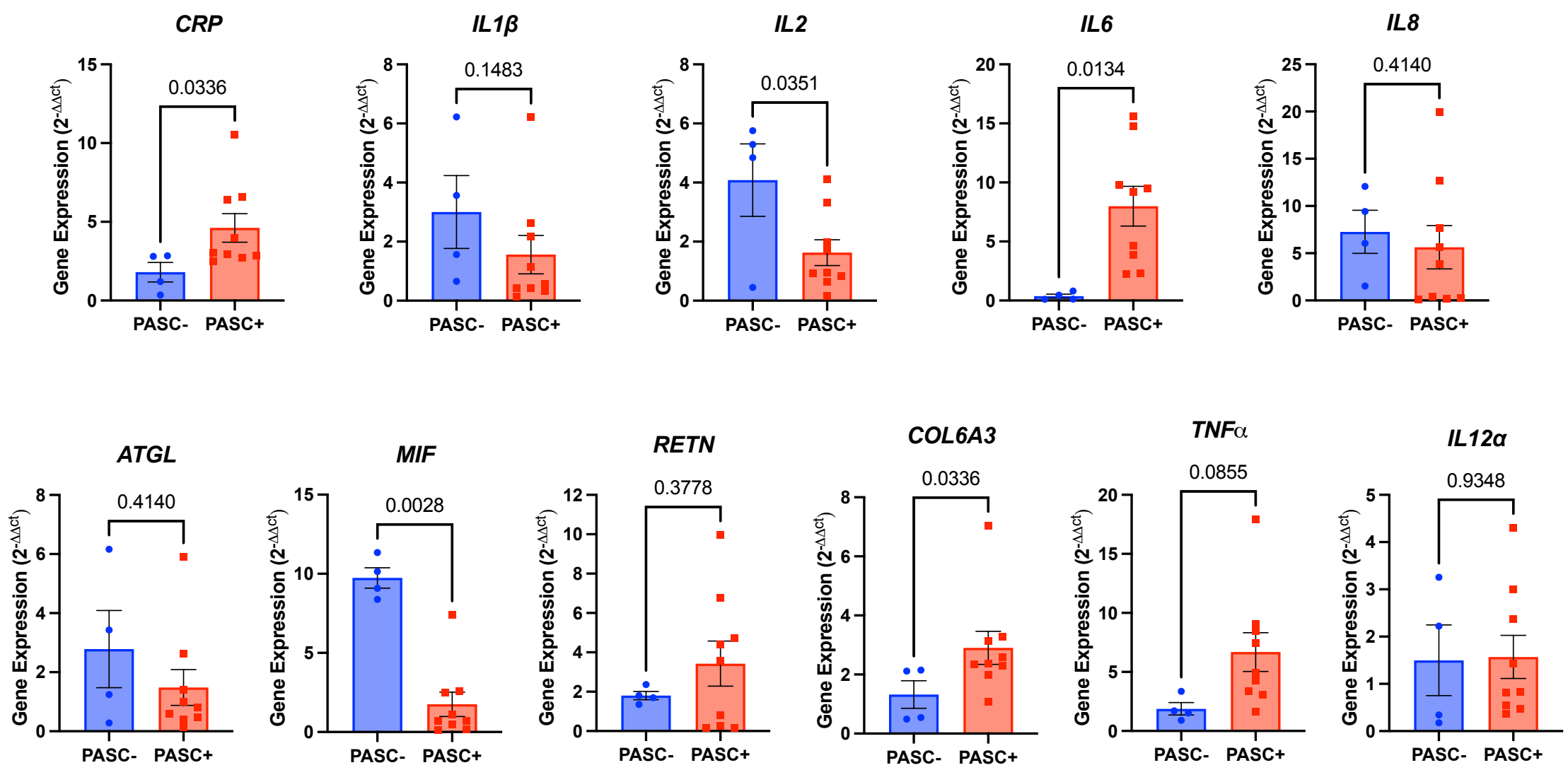

Supplement: Supplementary file 2 [file Image1.pdf]
