# Supplementary material for: Metabolic, viral, and immune phenotypes in long COVID
Source: Front Endocrinol (Lausanne). 2026 Aug 11;17:1899115. doi: 10.3389/fendo.2026.1899115 (PMC13508033; doi:10.3389/fendo.2026.1899115)
Supplement: Supplementary file 3 [file Table1.docx]

| **Supplemental Table 1. Demographics by PROMIS Scores** | | | | |
| --- | --- | --- | --- | --- |
| **Characteristics** | **HSB (n=25)**  **Mean ± Std** | **LSB (n=15)**  **Mean ± Std** | **ISB (n=14)**  **Mean ± Std** | **All (n=54)**  **Mean ± Std** |
| **Age** | 51.88 ± 15.23 | 50.4 ± 13.56 | 52.79 ± 11.06 | 51.7 ± 13.58 |
| **Sex** |  |  |  |  |
| Female | 21 | 10 | 11 | 42 |
| Intersex | 0 | 1 | 0 | 1 |
| Male | 4 | 3 | 3 | 10 |
| Unknown | 0 | 1 | 0 | 1 |
| **Race** |  |  |  |  |
| White | 19 | 12 | 12 | 43 |
| Black | 2 | 1 | 2 | 5 |
| Black/white | 2 | 0 | 0 | 2 |
| White/American Indian | 1 | 0 | 0 | 1 |
| White/Middle Eastern or North African | 1 | 1 | 0 | 2 |
| missing | 0 | 1 | 0 | 1 |
| **Ethnicity** |  |  |  |  |
| Hispanic | 2 | 0 | 0 | 2 |
| Not Hispanic/Latino | 23 | 15 | 14 | 52 |
| **Vaccination status** |  |  |  |  |
| Yes | 23 | 14 | 13 | 50 |
| No | 2 | 1 | 1 | 4 |
| **Time post infection, days** | 1040.56 ± 301.99 | 783.67 ± 249.46 | 753.14 ± 184.53 | 894.69 ± 291.38 |
| **Weight, lbs** | 195.56 ± 42.04 | 172.49 ± 33.2 | 198.18 ± 46.14 | 189.83 ± 41.62 |
| **BMI, kg/m^2^** | 32.13 ± 6.78 | 28.65 ± 3.89 | 31.59 ± 7.53 | 31.02 ± 6.4 |
| **% Fat Mass** | 41.07 ± 10.31 | 38.89 ± 5.67 | 42.57 ± 9.47 | 40.86 ± 8.97 |
| **% Lean Mass** | 53.93 ± 5.14 | 58.39 ± 5.33 | 55.11 ± 8.85 | 55.48 ± 6.5 |
| **Femoral BMD, g/cm^2^** | 0.88 ± 0.12 | 0.97 ± 0.1 | 0.96 ± 0.17 | 0.93 ± 0.13 |
| **Spine BMD, g/cm^2^** | 1.15 ± 0.15 | 1.26 ± 0.14 | 1.16 ± 0.14 | 1.18 ± 0.15 |
| **Whole Body BMD, g/cm^2^** | 1.18 ± 0.15 | 1.19 ± 0.15 | 1.17 ± 0.14 | 1.18 ± 0.14 |

| **Supplemental Table 2. Clinical Phenotypes by LCRI Classification** | | | |
| --- | --- | --- | --- |
| **Phenotype** | **Likely Long COVID(LC)(n=15)** | **Indeterminate**  **(n=39)** | ***P*-value**  **(LC vs Indeterminate)** |
| **Cholesterol, mg/dL** | 220.53 ± 9.71 | 193.34 ± 6.10 | 0.02 |
| **LDL cholesterol, mg/dL** | 130.65 ± 8.76 | 106.46 ± 5.43 | 0.02 |
| **Leptin, ng/mL** | 41.0 ± 6.5 | 26.6 ± 4.14 | 0.08 |
| **HOMA-B** | 227.95 ± 47.60 | 101.21 ± 26.51 | 0.03 |
| **HOMA-IR** | 4.16 ± 0.85 | 2.36 ± 0.47 | 0.07 |
| **Insulin, AUC** | 12643 ± 1838 | 8708 ± 1098 | 0.07 |
| **Left Femoral Neck BMD, g/cm^2^** | 0.86 ± 0.04 | 0.95 ± 0.03 | 0.08 |
| **CD14, cells/mm^3^** | 0.81 ± 0.10 | 0.56 ± 0.07 | 0.05 |
| **CD66b, cells/mm^3^** | 0.81 ± 0.18 | 1.31 ± 0.13 | 0.03 |
| **CD3/CD26** | 59.48 ± 3.33 | 46.38 ± 2.32 | 0.002 |
| **CD26 activity** | 16.13 ± 1.43 | 9.94 ± 0.99 | 0.001 |
| **Calcium, mg/dL** | 9.67 ± 0.10 | 9.43 ± 0.06 | 0.04 |
| **PTT** | 32.27 ± 0.77 | 27.92 ± 0.48 | <0.0001 |
| Data presented as mean ± standard error | | | |

| **Supplemental Table 2A. Clinical Phenotypes by PROMIS Classification with Effect Size** | | | | |
| --- | --- | --- | --- | --- |
| **Phenotype** | **HSB (n=25)**  **Mean ±SE** | **LSB (n=15)**  **Mean ±SE** | ***P*-value (HSB vs LSB)**  **Mean ±SE** | **Effect size** |
| **Leptin, ng/mL** | 40.2 ± 4.8 | 18.3 ± 6.1 | 0.007 | -0.343 |
| **% Lean Mass** | 53.93 ± 1.27 | 58.39 ± 1.64 | 0.035 | 0.290 |
| **Spine BMD, g/cm^2^** | 1.15 ± 0.04 | 1.26 ± 0.05 | 0.073 | 0.346 |
| **R.Femoral BMD, g/cm^2^** | 0.88 ± 0.03 | 0.99 ± 0.04 | 0.06 | 0.248 |
| **Total protein, g/dL** | 7.36 ± 0.08 | 7.09 ± 0.11 | 0.059 | 0.243 |
| **WBC, cells x 10^3^/μl** | 7.17 ± 0.35 | 5.79 ± 0.44 | 0.02 | -0.293 |
| **ETP, ng/mL** | 39.14 ± 4.90 | 25.39 ± 6.54 | 0.099 | -0.272 |
| **Cortisol, *µ*g/dL** | 8.79 ± 1.00 | 11.98 ± 1.46 | 0.08 | 0.326 |

| **Supplemental Table 2B. Clinical Phenotypes by LCRI Classification with Effect Size** | | | |
| --- | --- | --- | --- |
| **Phenotype** | **Likely Long COVID(LC)(n=15)** | **Indeterminate**  **(n=39)** | **Effect size** |
| **Cholesterol, mg/dL** | 220.53 ± 9.71 | 193.34 ± 6.10 | -0.343 |
| **LDL cholesterol, mg/dL** | 130.65 ± 8.76 | 106.46 ± 5.43 | -0.323 |
| **Leptin, ng/mL** | 40.50 ± 6.50 | 26.6 ± 4.14 | -0.258 |
| **Left Femoral Neck BMD, g/cm^2^** | 0.86 ± 0.04 | 0.95 ± 0.03 | 0.303 |
| **Calcium, mg/dL** | 9.67 ± 0.10 | 9.43 ± 0.06 | -0.289 |
| **PTT** | 32.27 ± 0.77 | 27.92 ± 0.48 | -0.659 |
